# Kidney-transplant patients receiving living- or dead-donor organs have similar psychological outcomes (findings from the *PI-KT* study)

**DOI:** 10.1108/MIJ-10-2019-0002

**Published:** 2020-02-20

**Authors:** Helge H.O. Müller, Caroline Lücke, Matthias Englbrecht, Michael S. Wiesener, Teresa Siller, Kai Uwe Eckardt, Johannes Kornhuber, J. Manuel Maler

**Affiliations:** Department of Psychiatry and Psychotherapy, University Hospital Bonn, Bonn, Germany; Department of Medical Psychology, University Hospital Bonn, Bonn, Germany and Department of Psychiatry and Psychotherapy, Friedrich-Alexander-Universität Erlangen-Nürnberg, Erlangen, Germany; Department of Rheumatology, Friedrich-Alexander-Universitat Erlangen-Nurnberg, Erlangen, Germany; Department of Nephrology, Friedrich-Alexander-Universitat Erlangen-Nurnberg, Erlangen, Germany; Department of Psychiatry and Psychotherapy, Friedrich-Alexander-Universitat Erlangen-Nurnberg, Erlangen, Germany; Department of Nephrology, Charite Medical Faculty Berlin, Berlin, Germany; Department of Psychiatry and Psychotherapy, Friedrich-Alexander-Universitat Erlangen-Nurnberg, Erlangen, Germany

**Keywords:** Coping, Kidney transplantation

## Abstract

**Purpose:**

Kidney transplantation (KT) is the treatment of choice for end-stage chronic kidney disease (CKD) and is well known to improve the clinical outcome of patients. However, the impact of KT on comorbid psychological symptoms, particularly depression and anxiety, is less clear, and recipients of living-donor (LD) organs may have a different psychological outcome from recipients of dead-donor (DD) organs.

**Design/methodology/approach:**

In total, 152 patients were included and analyzed using a cross-sectional design. Of these patients, 25 were pre-KT, 13 were post-KT with a LD transplant and 114 were post-KT with a DD transplant. The patients were tested for a variety of psychometric outcomes using the Hospital Anxiety and Depression Scale, the 12-Item Short Form Health Survey (assessing physical and mental health-related quality of life), the Resilience Scale, the Coping Self-Questionnaire and the Social Support Questionnaire.

**Findings:**

The mean age of the patients was 51.25 years and 40 per cent of the patients were female. As expected, the post-KT patients had significantly better scores on the physical component of the Short Form Health Survey than the pre-KT patients, and there were no significant differences between the two post-KT groups. There were no significant differences among the groups in any of the other psychometric outcome parameters tested, including anxiety, depression and the mental component of health-related quality of life.

**Research limitations/implications:**

KT and the origin of the donor organ do not appear to have a significant impact on the psychological well-being of transplant patients with CKD. Although the diagnosis and early treatment of psychological symptoms, such as depression and anxiety, remain important for these patients, decisions regarding KT, including the mode of transplantation, should not be fundamentally influenced by concerns about psychological impairments at the population level.

**Originality/value:**

CKD is a serious condition involving profound impairment of the physical and psychological well-being of patients. KT is considered the treatment of choice for most of these patients. KT has notable advantages over dialysis with regard to the long-term physical functioning of the renal and cardiovascular system and increases the life expectancy of patients. However, the data on the improvement of psychological impairments after KT are less conclusive.

## List of abbreviations

CKD: = chronic kidney disease;
DD: = dead donor;
FKV: = Freiburger Fragebogen zur Krankheitsverarbeitung;
F-SozU: = Social Support Questionnaire;
HADS: = Hospital Anxiety and Depression Scale;
KT: = kidney transplantation;
LD: = living donor;
PROs: = patient-reported outcomes; and
RS: = resilience scale.

## Introduction

Chronic kidney disease (CKD) is a serious condition involving profound impairment of the physical and psychological well-being of patients. Kidney transplantation (KT) is considered the treatment of choice for most of these patients. KT has notable advantages over dialysis with regard to the long-term physical functioning of the renal and cardiovascular system and increases the life expectancy of patients (Schnuelle *et al.*, 1998; Sorensen *et al.*, 2016). However, the data on the improvement of psychological impairments after KT is less conclusive. The rates of depression and anxiety in CKD patients are considerably higher than those in general population (Halen *et al.*, 2012). Moreover, the rates of depression appear to remain high after successful KT (Chilcot *et al.*, 2014). Some studies comparing KT patients with patients on hemodialysis using the same study design have found lower rates of depression in KT patients than in patients on hemodialysis (Szeifert *et al.*, 2010; Cameron *et al.*, 2000), whereas others have found similar rates of depression and psychiatric distress in both groups of patients (Kalman *et al.*, 1983).

A better understanding of the characteristics of risk factors for and possible prevention of depression in CKD patients is important not only because of the obvious burden on the patient’s overall and, especially psychological, well-being but also because of the depressive symptoms that have a notable influence on patients’ physical outcome.

For example, a large prospective cohort study showed that health-related quality of life scores was associated with clinical outcomes in kidney-transplanted patients (Molnar-Varga *et al.*, 2011). Similarly, a recent meta-analysis of cohort studies showed a consistent link between depression and increased mortality in CKD (Palmer *et al.*, 2013).

In addition to various psychological symptoms, a substantial number of confounding factors are important to the understanding of the complex constellation in KT patients (Muller *et al.*, 2015; Griva *et al.*, 2002; Parsaei Mehr *et al.*, 2011; de Groot *et al.*, 2013; Cabral *et al.*, 2015; Zimmermann *et al.*, 2016).

In a recent cross-sectional study (psychiatric impairments in KT [PI-KT] study), we compared patients waiting for transplantation with kidney-transplanted patients regarding a variety of psychometric parameters and found no significant differences in depression, anxiety, resilience and health-related quality of life among the groups (Muller *et al.*, 2015). However, one limitation of that study was the inclusion of only cadaveric-transplanted patients. This limitation may be important because several studies have reported possible differences between recipients of living-donor (LD) transplantations and recipients of cadaveric-donor transplantations in various aspects of mental health, including anxiety and depression (Griva *et al.*, 2002; Parsaei Mehr *et al.*, 2011; de Groot *et al.*, 2013; Cabral *et al.*, 2015; Zimmermann *et al.*, 2016). To address the influence of the type of transplantation, in the present study, we explicitly included a group of patients with LD kidney transplants.

## Methods

### Study design and procedure

This cross-sectional study was conducted in cooperation with the Department of Nephrology and the Department of Psychiatry and Psychotherapy of the University Hospital Erlangen. The study groups included are part of the abovementioned examination of kidney-transplanted patients (the *PI-KT* study) (Muller *et al.*, 2015).

The clinical patient groups eligible to participate were as follows:

- patients with CKD before a planned KT (pre-KT);
- patients after KT with a LD organ (post-KT-LD); and
- patients after KT with a DD organ (post-KT-DD).

The study was evaluated and approved by the Ethics Committee of the University of Erlangen-Nuremberg. All patients received detailed information regarding the study design and the procedures and signed a written informed consent form before inclusion. Data acquisition for individual patients was performed on a single date, typically during a routine KT-protocol visit and, generally, took approximately 45 min for each proband, which is comparable to the duration of a normal psychiatric consultation.

The general somatic and nephrological comorbidity burdens of all probands were also collected.

### Patient-reported outcomes

The sociodemographic assessment included age, gender, marital status and educational level. The somatic parameters included information regarding the duration of dialysis treatment and a range of biochemical kidney functioning parameters. The psychiatric assessments were performed in either the form of patient self-report scales or rater-based assessments. In detail, the following psychometric scales were applied:

- The *Hospital Anxiety and Depression Scale (HADS-D/A)* (Snaith and Zigmond, 1986) is a validated, 14-item screening tool for the prevalence of depression and anxiety in hospital patients. Two seven-item subscales assess depressive and anxiety symptoms, generating scores of 0-21. Higher scores indicate a more pronounced clinical pathology with subscale sums ≥8 indicating clinically relevant depression/anxiety.
- The *Resilience Scale (RS)* (Wagnild and Young, 1993) is a 25-item tool designed to measure a person’s ability to mentally recover from negative life events, such as a severe illness. The concept of resilience can be divided into two factors, i.e. personal competence and acceptance of self and life, which are measured by the subscales RS-C and RS-A, respectively.
- The *Coping Self-Questionnaire* (Freiburger Fragebogen zur Krankheitsverarbeitung, FKV in German) (FA, 1989) comprises 102 items that can be divided into the following five subscales (FKVF 1-5): depressive coping; active, problem-focused coping; diversion and self-encouragement; religiousness and search for coherence and trivializing and wishful thinking, which were chosen because coping is linked to resilience and describes a patient’s adaptive or maladaptive strategies for coping with the impacts of negative life events.
- The *12-Item Short-Form Health Survey* (Ware *et al.*, 1996) is the short form of the SF-36 health profile and measures a person’s overall health as an indicator of health-related quality of life. The questionnaire covers a variety of qualities, such as limitations in daily activities, interference with social activities, the nonaccomplishment of goals due to health problems and the frequency of emotional states, such as feeling energetic, peaceful or downhearted. The ratings are divided into two components, “Physical” (PCS-12) and “Mental” (MCS-12) health, with scores ranging from 0 to 100.
- The *Social Support Questionnaire, short form (F-SozU)* (German title: Fragebogen zur sozialen Unterstützung) (Fydrich *et al.*, 2009) is a validated questionnaire consisting of 14 items that are rated on a five-point Likert scale that assesses the perceived and anticipated social support that a patient receives from his/her social environment.

### Data processing and analysis

The data were collected and saved in an anonymized form. The data analysis was performed using SPSS 21 (IBM Corp., Armonk, NY). The descriptive data analysis was performed for all demographic data, somatic data and patient-reported outcomes (PROs). The descriptive data are shown as the median [interquartile range] unless otherwise indicated. The data from the psychometric questionnaires were inspected for normality of distribution using both numerical and graphical methods (Shapiro–Wilk test and Q-Q plots). Because the data from some questionnaires were not normally distributed, we used nonparametric statistics. Potential differences among the pre-KT, KT-LD and KT-DD patient groups were tested using the Kruskal–Wallis test for interval data, and differences in frequency distributions (i.e. nominal data) were tested using the *χ*^2^-test. For significant results in the Kruskal–Wallis analysis, post hoc comparisons were calculated using the Mann–Whitney U-test with Bonferroni–Holm adjustment for multiple comparisons. The level of significance was set to *p* ≤ 0.05.

## Results

### Study sample and demographics

For this analysis, we considered a total of 342 CKD patient visits, excluding evaluations with missing data on the type of donor organ (*N* = 118), multiple assessments of CKD patients over time, and patients who had experienced a failed KT and were waiting a new transplant (*N* = 8). Accordingly, our final data set included 152 patients. In total, 59.9 per cent of the patients were males and 40.1 per cent were females. The mean ± standard deviation age was 51.25 ± 15.12 years. Among these patients, 25 were pre-KT, 13 were post-KT-LD and 114 were post-KT-DD.

Demographic characteristics, including age, gender, relationship status, educational status and duration of dialysis, are shown in Table I. The distributions of relationship status, educational level, employment status and psychoactive medication did not differ significantly among the groups. The Kruskal–Wallis test showed between-group differences in age (*p* = 0.014) and duration of dialysis (*p* = 0.001). Post hoc testing showed that the post-KT-DD patients were older than the pre-KT patients (*p* = 0.004) (pre-KT = 43.3[27.0] and post-KT-DD = 52.8[22.6]). No significant age differences were identified between post-KT-LD patients and those in the other two groups. All three subgroups differed from one another in the duration of dialysis (prior to KT) (pre-KT vs post-KT-LD: *p* = 0.002; pre-KT vs post-KT-DD: *p* = 0.002 and post-KT-LD vs post-KT-DD: *p* = 0.04) with the shortest period of dialysis in the pre-KT groups (10.00[35.0]) followed by the post-KT-DD (54.00[79.0]) and post-KT-LD groups (117.0[160.0]). All of the aforementioned significant post hoc tests remained significant after Bonferroni–Holm adjustment.

### Patient-reported outcomes

The mean scores obtained on the various psychometric questionnaires are shown in Table II. The Kruskal–Wallis test showed a statistically significant difference among the groups in the SF-12 physical component summary scale (*p* = 0.007), whereas only the post hoc test for pre-KT (53.44[6.1]) vs post-KT-DD (44.69[7.1]) patients remained significant (*p* = 0.002) after adjustment, suggesting reduced physical well-being among post-KT-DD patients compared to that among the pre-KT subgroup. No significant difference was found in the SF-12 physical component scale between the post-KT-LD and post-KT-DD groups. Additionally, a cluster analysis showed no significant differences in the nephrological disease that led to KT (e.g. hypertensive origin, immunological associated and analgesics associated) but was carefully interpreted with regard to the sample sizes.

Furthermore, no significant differences were found among the three subgroups of patients in any of the other PROs. The corresponding *p*-values of the comparisons of each questionnaire are shown in Table II.

Regarding the symptoms of depression and anxiety, the mean HADS total scores and the anxiety and depression subscale scores were similar among the groups, ranging from 4 to 6. The rate of patients scoring above the cut-off value for clinically relevant depression/anxiety on the HADS (i.e. corresponding HADS subscale score ≥ 8) depression and anxiety scales was not related to patient subgroup according to the *χ*^2^-test (*p*_Depression_ = 0.20, *p*_Anxiety_ = 0.83).

## Discussion

The results of the present study are consistent with our recent finding that psychiatric symptoms, including depression, anxiety and health-related quality of life, did not significantly change after KT compared to those of the pre-KT state (Muller *et al.*, 2015). However, the overlap in the study populations between both studies must be considered. In contrast to the previous study, this study compared DD and LD organ recipients, showing no significant differences in the psychological outcome parameters. There was a significant difference in the mean scores on the SF-12 physical component summary scale, and the post-KT patients in both donor groups showed fewer physical limitations than the pre-KT group. This result is an expected finding that is consistent with the well-known benefits of KT on physical health (Halen *et al.*, 2012).

In contrast to previous studies, our study shows no differences between the two modes of transplantation in depression, anxiety, resilience, coping, health-related quality of life and social support, indicating that the origin of the donor organ plays a negligible role in the psychological outcome of the patients. The question of whether different emotional responses of transplanted patients depend on the origin of the donor organ has been a topic of controversy in the recent literature. Griva *et al.* (2002), who included a comparable group size (347 probands, 76 LDs and 271 DDs), found that LD recipients expressed more guilt toward the donor than the DD recipients, but the levels of health-related quality of life did not differ between the groups. Parsaei Mehr *et al.* (2011) compared LD and DD organ recipients (60 DDs and 60 LDs) with regard to depression and anxiety and found significant differences with the LD recipients suffering from increased levels of both depression and anxiety. Similarly, a recent study by Zimmermann *et al.* (2016) noted possible psychological disadvantages for LD recipients (68 LDs and 173 DDs were examined), with this group showing both more guilt toward the donor and higher rates of clinically significant anxiety. In contrast, the Patients After Renal Transplantation and donation: long-term Effects on Health and Relationship study showed better outcomes in LD recipients than in DD recipients in some domains of health-related quality of life and social participation (de Groot *et al.*, 2013).

The results of this study indicate that neither the emotional responses of depression and anxiety nor the health-related quality of life are substantially influenced by the origin of the donor organ. This finding may be clinically important because concerns regarding a possible negative impact of either type of donor, as suggested in the literature, may lead to insecurities during transplantation procedures and affect patients’ donor-organ acceptance, potentially even leading to rejection of suitable organs. Therefore, the findings of this study suggest that concerns regarding a possible psychological burden imposed by donor organ transplantation should not play a major role in the decision-making process for KT compared to other important factors, such as organ availability, waiting time, reduced risk of graft failure with LD organs (Roodnat *et al.*, 2003) and the potential health risk to LDs in LD-KT.

Nevertheless, the high prevalence of depression and anxiety in patients with chronic kidney failure, independent of the transplantation type and the somatic reason for the transplantation, continues to be an important problem that requires effective measures to ensure an early diagnosis and adequate psychiatric treatment.

Some relevant psychological aspects of LD transplantations were not assessed by the psychometric questionnaires used in our study, including the expression of feelings of guilt regarding the donor, which was found to play a role in previous studies, and the possible negative or positive changes in the relationships between the donors and the recipients (de Groot *et al.*, 2012). In particular, complex psychological paradigms, such as shame and guilt are difficult to measure with the validated instruments used in comorbid somatically/psychological probands. It seems that (in probands that are not somatically ill), shame is more strongly associated with anxiety symptoms than guilt. Our study sample was too small to use variance analyses to control for sub-symptoms concerning the influence of guilt and shame on KT in KD/DD. Upcoming larger samples should control for those potentially influencing factors, especially in KT probands suffering from comorbid anxiety symptoms.

Nonetheless, in cases of potentially negative dynamics between the donor and the recipient, other psychometric scores are likely to show a strong impact as well, particularly depression scores. Thus, although these factors may play a significant role in single cases, there are no indications of a strong influence on overall psychological well-being at the population level.

The present study has some limitations. First, the number of patients included in each group differed considerably, with comparably small sample sizes in the pre-KT and post-KT-LD groups. Because the sample sizes were dictated by the cross-sectional study design, this aspect can also be regarded as a study result. However, to improve the level of confidence in the results, future studies should examine larger patient populations, particularly in the LD KT group, preferably in a prospective design. Additionally, the somatic aspects of the disease that led to dialysis and KT must be controlled in larger study samples.

A significant age difference was found between the pre-KT and post-KT-DD patient groups. However, this age difference was 10 years and does not cover typical life phases of altered psychiatric vulnerability, such as the transition from working life to retirement. Our main finding of no psychometric differences between LD and DD transplanted patients is unlikely to be biased by age-related factors.

## Conclusions

Our results do not support the theory that the origin of a donor organ has a major or significant influence on the psychological outcome of kidney-transplanted patients, and they imply that concerns regarding psychological factors should not play a large role in decision-making regarding the type of transplantation in end-stage kidney disease.

## Declaration Statements

### Ethics approval and consent to participate

This study was reviewed and approved by the ethics committee of the Friedrich-Alexander University Erlangen-Nuremberg. All probands provided informed written consent for participation in the study and publication of this work. In addition to registration with the local ethics committee, the study is also retrospectively registered.

### Funding

The study received intramural funding from the Department of Psychiatry of the Friedrich-Alexander-University Erlangen-Nuremberg (Head of Department: Prof Dr Johannes Kornhuber) and within the funding program *ELAN* (Erlanger Leistungsbezogene Anschubfinanzierung).

### Acknowledgements

None

### Consent for publication

Consent for publication was obtained from all probands of this study.

### Availability of data and materials

All data are presented in the manuscript. There are no additional data.

### Authors’ contributions

H.H.O.M., J.M.M. and J.K. designed the study. H.H.O.M., T.S. and M.S.W. participated in data acquisition. H.H.O.M. and C.L. wrote the manuscript (contributed equally). C.L. and M.E. provided expert opinions on the statistical procedures and analyzed the data. M.S.W. and K.U.E. provided expert opinions on the nephrology and transplantation procedures. J.K. and A.P. advised regarding the psychometric measurements of anxiety and affective disorders. H.H.O.M., T.S., J.K., K.U.E. and J.M.M. provided expert advice on the diagnoses of psychiatric disorders in somatically ill probands. J.M.M. was the senior author and critically revised the versions of the manuscript. All authors participated in writing and revising the final manuscript.

### Competing interests

All authors declare that they have no competing interests.

## Figures and Tables

**Table I:** Demographic data of the participants

	Groups and mode of transplantation									
	**Pre-KT (KT planned)**			Post-KT (living donor)			Post-KT (dead donor)			
	Median (IQR)	*N*	Column, *N* (%)	Median (IQR)	*N*	Column, *N* (%)	Median (IQR)	*N*	Column, *N* (%)	*p*-value
***N***		25			13			114		
**Age**	43.3 (27.0)			53.5 (20.1)			52.8 (22.6)			0.014*
**Single**		7	28.0		1	9.1		39	36.4	0.157
**In a relationship**		18	72.0		10	90.9		68	63.6	
**No. of children**	1.48			1,0			1,35			0.564
**Up to basic primary school**		5	20.8		3	27.3		27	25.7	0.953
**High school diploma**		12	50.0		4	36.4		46	43.8	
**University-entrance diploma**		7	29.2		4	36.4		32	30.5	
**Duration of dialysis (months)**	10.0 (35.0)			117.0 (160.0)			54.0 (79.0)			0.001*
**Working full time**		7	29.2		2	16.7		25	22.5	0.354
**Working part time**		5	20.8		2	16.7		13	11.7	
**Not working**		2	8.3		0	0.0		9	8.1	
**Unemployed**		3	12.5		0	0.0		5	4.5	
**Receiving pension**		7	29.2		8	66.7		59	53.2	

Note: *Statistically significant between-group differences (*p* ≤* 0.05)

**Table II:** Patient-reported outcome results

	Pre-KT	Post-KT living donor	Post-KT dead donor	Significance level
	Median (IQR)	Median (IQR)	Median (IQR)	*p*-value
**SF-12 physical component scale (PCS-12)**	53.44 (6.09)	45.13 (10.13)	44.69 (7.12)	0.007*
**SF-12 mental component scale (MCS-12)**	42.12 (9.20)	51.73 (12.14)	46.36 (12.73)	0.465
**HADS total score**	4.0 (5.0)	5.5 (5.5)	4.5 (5.0)	0.713
**HADS-A (anxiety)**	5.0 (6.0)	6.0 (6.0)	4.0 (5.0)	0.512
**HADS-D (depression)**	4.0 (6.0)	5.0 (5.0)	4.0 (4.0)	0.959
**Resilience scale-competence**	101.0 (17.0)	98.0 (28.0)	100.0 (20.0)	0.691
**Resilience scale-acceptance**	48.0 (14.0)	47.0 (15.0)	47.0 (11.0)	0.641
**Resilience scale-total score**	148.0 (32.0)	146.0 (45.0)	147.0 (27.0)	0.619
**Depressive coping (FKVF 1)**	2.2 (1.0)	2.2 (1.4)	2.0 (1.0)	0.226
**Active, problem-focused coping (FKVF 2)**	3.6 (0.8)	3.6 (0.6)	3.8 (1.4)	0.597
**Diversion and self-encouragement (FKVF 3)**	3.2 (0.8)	3.0 (0.7)	3.4 (1.2)	0.694
**Religiousness and search for coherence (FKVF 4)**	2.8 (1.4)	3.0 (1.2)	2.9 (1.1)	0.715
**Trivializing and wishful thinking (FKVF 5)**	2.0 (0.7)	2.0 (1.0)	2.0 (1.3)	0.989
**FKV-total score**	2.7 (0.7)	2.9 (0.4)	2.8 (0.7)	0.909
**Social Support Questionnaire-total score**	1.64 (0.71)	1.50 (0.82)	1.57 (0.79)	0.656

Note: *Statistically significant difference (*p* ≤ 0.05)
